# An Efficient Method for Laser Welding Depth Determination Using Optical Coherence Tomography

**DOI:** 10.3390/s23115223

**Published:** 2023-05-31

**Authors:** Guanming Xie, Sanhong Wang, Yueqiang Zhang, Biao Hu, Yu Fu, Qifeng Yu, You Li

**Affiliations:** 1 Institute of Intelligent Optical Measurement and Detection, Shenzhen University, Shenzhen 518060, China; xieguanming2020@email.szu.edu.cn (G.X.);; 2 College of Physics and Optoelectronic Engineering, Shenzhen University, Shenzhen 518060, China; 3 Shenzhen Sincevision Technology Co., Ltd., Shenzhen 518055, China; 4 National Key Laboratory of Human Factors Engineering, China Astronaut Research and Training Center, Beijing 100094, China

**Keywords:** laser welding, optical coherence tomography, welding depth, outlier, DBSCAN, percentile filter

## Abstract

Online monitoring of laser welding depth is increasingly important, with the growing demand for the precise welding depth in the field of power battery manufacturing for new energy vehicles. The indirect methods of welding depth measurement based on optical radiation, visual image and acoustic signals in the process zone have low accuracy in the continuous monitoring. Optical coherence tomography (OCT) provides a direct welding depth measurement during laser welding and shows high achievable accuracy in continuous monitoring. Statistical evaluation approach accurately extracts the welding depth from OCT data but suffers from complexity in noise removal. In this paper, an efficient method coupled DBSCAN (Density-Based Spatial Clustering of Application with Noise) and percentile filter for laser welding depth determination was proposed. The noise of the OCT data were viewed as outliers and detected by DBSCAN. After eliminating the noise, the percentile filter was used to extract the welding depth. By comparing the welding depth determined by this approach and the actual weld depth of longitudinal cross section, an average error of less than 5% was obtained. The precise laser welding depth can be efficiently achieved by the method.

## 1. Introduction

Laser welding was widely used in the field of the power battery manufacturing for new energy vehicles for its benefits such as high energy density, small heat affected zone and fast welding speed. In the manufacturing of power battery, uncontrolled laser welding depth gives rise to the risk of the piercing of the battery cell, with subsequent leaking of harmful gases and fire [1]. For the quality assurance during laser welding, a precise online measurement of the welding depth is required.

Several methods based on indirect observation of optical radiation [2], visual image [3] and acoustic signals [4] were proposed to monitor the welding depth. The accuracy of these methods are easily affected by the process or material parameter changes and continuous monitoring of the welding depth could not be achieved with high-precision. Compared to the indirect methods, the optical coherence tomography (OCT), with the advantages of being fast, high-resolution and non-destructive, provides a direct welding depth measurement and shows great potential for laser welding monitoring.

The OCT is a well-established imaging technique in medical applications since the early 1990s [5], which is based on low-coherence interferometry. Spectral-domain OCT (SD-OCT) can measure small deviations in sample distances relative to a reference distance by recording interference pattern in a spectrally resolved way. This can be achieved by using a broadband light source as the radiation source and a spectrometer as the detector. SD-OCT has significant advantages of imaging speed and sensitivity and can be applied to non-medical applications [6,7]. In recent years, it showed promise in laser materials processing such as laser drilling [8], laser additive manufacturing [9,10,11] and laser welding [12,13,14,15].

In laser deep-penetration welding, a metal vapor capillary called keyhole is formed in the melt pool, enabling high aspect ratio of the weld seam. According to [16], the deviation between keyhole depth and welding depth is only 9 μm, which means that the welding depth can be indicated by the keyhole depth. By integrating the measuring beam into a laser welding head, OCT coaxially measures the keyhole depth through the interference between beams backscattered from the keyhole bottom and the reference arm. Webster et al. showed that the inline coherent imaging, which is based on SD-OCT, is capable of directly measuring the keyhole depth in a process as violent as kW-class laser welding [12]. The great advantage of this measurement method is that the OCT measuring beam is not influenced by the emitted process radiation, which could disturb those indirect methods. Kogel-Hollacher et al. also developed a measuring system based on SD-OCT to monitoring the keyhole depth. Via feeding the laser source with a control signal determined by the measured keyhole depth, closed-loop control of the welding depth was achieved [15].

**Figure 1:**
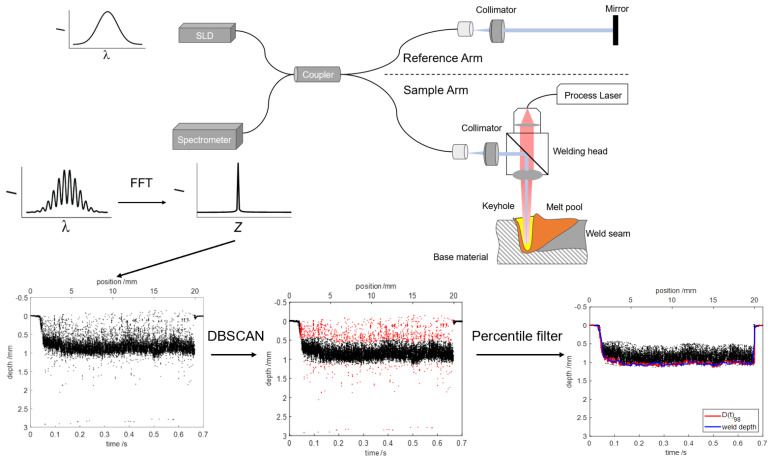
Laser welding depth measuring system using SD-OCT and two-step welding depth extraction method based on DBSCAN and percentile filter. The black points show the OCT measurements. The red points mark the detected noise.

Figure 1 shows the typical setup of laser welding depth measuring system using SD-OCT. Fiber coupled light from superluminescent diode (SLD) is split by a coupler into sample arm and reference arm. Light in the sample arm is superimposed with the processing laser beam via a dichroic beam splitter in welding head. Ideally, the imaging light hits the bottom of keyhole and is reflected back to the sample arm fiber. Light in the reference arm is back-reflected off a mirror and collected by the reference arm fiber. Light reflected from both arms is coupled into the coupler and transmitted to the spectrometer. Interference spectrogram is recorded by line-scan camera in the spectrometer. The depth information, called A-scan, is retrieved from the interference spectrogram by performing fast Fourier transform (FFT). The raw OCT data are obtained by tracking every A-scan.

The fluctuation of keyhole is generated by the process variations during laser welding, resulting in the dynamic changes in the size and shape of the keyhole. The raw OCT data contain not only signals from the keyhole bottom but also noise from the keyhole wall or the multiple reflection inside the keyhole, which cannot reveal the actual weld depth directly [17]. Therefore, post-processing of the raw OCT data is required to extract the welding depth.

A percentile filter was applied to the raw OCT data and showed the effectiveness of lessening minimal deviation between the extracted welding depth and the actual weld depth [18,19,20]. Due to the different signal densities, the filter parameters have to be adjusted to the process and measurement conditions. Boley et al. proposed a two-step statistical evaluation approach, which included a first step to segment the noise based on the Poisson distribution and a second step to extract the welding depth from the raw OCT data by applying the percentile filter [21]. This improved the accuracy of OCT measurement with an error of less than 5% and eliminated the need to adapt the filter parameters. However, the noise removal step requires binning the raw OCT data into ~0.2 mm by 5 ms or larger segments and is quite complicated and time-consuming. Here, we proposed an efficient method coupled DBSCAN (Density-Based Spatial Clustering of Applications with Noise) and percentile filter for laser welding depth determination using OCT. Before applying percentile filter, a density-based outlier detection approach DBSCAN was applied to the OCT data for eliminating the noise, as shown in Figure 1. Compared with the previous statistical evaluation approach, the proposed method removes the noise more simply and extracts the welding depth with high-precision. Moreover, we conducted the repeated experiment and verified the effectiveness of the proposed method. 

The rest of this paper is organized as follows. Section 2 gives a brief review on the principle of laser welding depth measurement using SD-OCT and a detailed description of the proposed welding depth extraction method. Section 3 shows the experimental results to verify the feasibility and robustness and Section 4 presents the conclusion.

## 2. Materials and Methods

### 2.1. Laser Welding Depth Measurement Using SD-OCT

Laser welding can be classified into two mechanisms: heat-conduction welding and deep-penetration welding. In heat-conduction welding, the laser power density is insufficient to vaporize metal materials. The laser is absorbed only at the surface of metal materials, resulting in broad and shallow weld seam with an aspect ratio smaller than 1. In deep-penetration welding, the laser power density is sufficiently high enough to cause vaporization by the presence of keyhole. Deep-penetration welding is widely used since it produces narrow and deep weld seam with an aspect ratio more than 1. Laser welding depth measurement using SD-OCT can be applied to the deep-penetration welding of metal materials (e.g., steel, aluminum, copper). The threshold value that describes the welding mode transition from heat-conduction to deep-penetration welding can be characterized either by laser power density (P/S) or by the ratio between laser power and laser spot diameter (P/d). According to [22], the P/d is more suitable than the P/S for describing the deep-penetration welding threshold in 1 μm-laser welding and the threshold of steel for deep-penetration welding is 1.5 kW/mm.

According to the theory of low-coherence interferometry, the detected interference spectrogram can be written as [23]

(1) $$I\left(k\right)=S\left(k\right)\left[R_{r}+\sum_{i=1}^{n}R_{i}+2\sqrt{R_{r}}\sum_{i=1}^{n}\sqrt{R_{i}}\cos\left(2k\Delta l_{i}\right)+\sum_{i\neq j=1}^{n}\sqrt{R_{i}R_{j}}\cos\left(2k\Delta l_{ij}\right)\right]$$

where $S\left(k\right)$ is the power spectrum of the light source, n is the number of the reflectors, $\Delta l_{i}$ is the path length difference between the i reflector and the reference arm and $\Delta l_{ij}$ is the path length difference between the i and j reflectors. The first two terms in the bracket are DC terms and the last term is autocorrelation term which describes the interference within the sample arm. These terms are the noise of SD-OCT and can be subtracted in the data processing. The third term represents the interference between light reflecting from sample and reference arms and encapsulates the axial depth information of the sample.

In laser welding depth measurement, light in the sample arm is reflected by the bottom of the keyhole which can be regarded as a single reflector. The detected interference spectrogram can be simplified as

(2) $$I\left(k\right)=s\left(k\right)\left[R_{r}+R_{1}+2\sqrt{R_{r}}\sqrt{R_{1}}\cos\left(2k\Delta l_{1}\right)\right]$$

where $\Delta l_{1}$ is the path length difference between the reflection location of keyhole bottom and the reference arm. The first two DC terms can be subtracted in the SD-OCT data processing steps. According to the Fourier transform (*FT*) pair $\frac{1}{2}\left[\delta \left(z+z_{0}\right)+\delta \left(z-z_{0}\right)\right]\overset{FT}{↔}\cos\left(kz_{0}\right)$ and the convolution property of *FT* $x\left(z\right)⊗y\left(z\right)\overset{FT}{↔}X\left(k\right)Y\left(k\right)$, the inverse *FT* of the interference spectrogram can be written as

(3) $$\left|FT_{k\to z}^{-1}\left[I\left(k\right)\right]\right|=\Gamma \left(z\right)⊗\left[\sqrt{R_{r}}\sqrt{R_{1}}\delta \left(z\pm 2\Delta l_{1}\right)\right]$$

where $\Gamma \left(z\right)$ is the *FT* of $S\left(k\right)$ and represents the envelope of the coherence function. The output of the convolution reveals the A-scan and the keyhole depth as a function of time can be obtained by tracking the $\Delta l_{1}$ of every A-scan.

### 2.2. Experimental Setup

**Figure 2:**
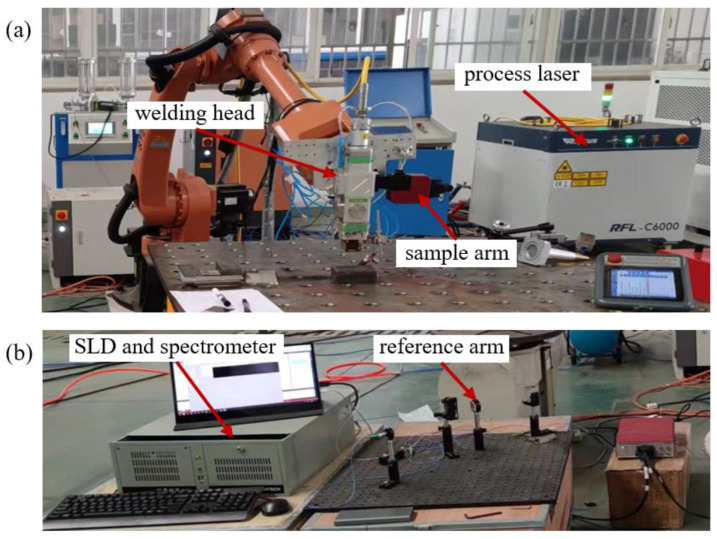
(**a**) Laser welding system; (**b**) SD-OCT system.

The experiments were conducted using a robot-based fixed optics system. The experimental setup of laser welding system and SD-OCT system are shown in Figure 2. A 6 kW multimode fiber laser with a wavelength of 1080 nm was used as process laser. The focal position of the processing beam was on the surface of the material with a focus diameter of 400 μm. The P/d was 2 kW/mm, resulting in deep-penetration welding mode. Mild steel contains approximately 0.05–0.30% carbon and is widely used in industry because of its good mechanical properties. In the laser welding experiment, 4 mm thick Q235 mild steels were used. The chemical composition of Q235 mild steel is shown in Table 1. Argon was applied as the shielding gas.

Single mode fiber-coupled light from SLD with a mean wavelength of 850 nm and a bandwidth of 45 nm was split by a coupler into reference arm and sample arm. Light in the sample arm was collimated by a collimator installed in a custom optical mount and aligned coaxially with the processing beam using the dichroic beam splitter in welding head. This light was focused by a focusing lens into a 54 μm spot size (1/e^2^). Light in the reference arm passed through polarization controller (PC) and dispersion compensator (DC) to correct for polarization changes caused by the single mode fiber and dispersion mismatch caused by the optics in the sample arm. The interference spectrogram was recorded on the spectrometer by a high-speed CMOS line-scan camera at 80 kHz with a sample of 2048 pixels.

Typical SD-OCT data processing steps include DC subtraction, linear wave number interpolation, spectral shaping and FFT to yield A-scan. The theoretical axial resolution of the system was ~7 μm, which was defined by the coherence length

(4) $$l_{c}=\frac{2\ln2}{\pi }\frac{\lambda^{2}}{\Delta \lambda }$$

where λ is the center wavelength and Δλ is the bandwidth of the light source. The experimental axial resolution was ~17 μm by measuring the full width at half maximum (FWHM) of the largest peak of A-scan, which was mainly limited by the imperfect dispersion matching between sample and reference arms.

### 2.3. Analysis of the Raw OCT Data

**Figure 3:**
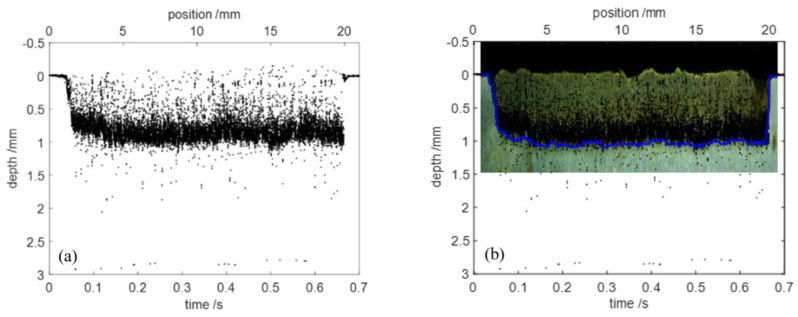
(**a**) Raw OCT data for laser welding. (**b**) Comparison of the raw OCT data and the longitudinal cross section of the welded seam (micrograph). The blue line marks the actual weld depth, which is extracted from the longitudinal cross section.

The keyhole depth as a function of time can be tracked by searching the brightest pixel of every A-scan. A threshold was set to remove the weak signals of little confidence and a pre-scan surface was subtracted from the OCT data. A typical plot of the raw OCT data that was acquired from a laser welding process in 4 mm thick mild steel with a welding speed of v = 30 mm/s and P/d = 2 kW/mm is shown in Figure 3a. Every black point represents an effective OCT depth measurement at the given time. The laser was turned on 0.03 s after beginning the measurement, and the surface was measured during the first 0.03 s. With the keyhole opened by the laser, the keyhole depth was measured until the laser was turned off at 0.66 s. The surface was measured again for the remaining measurement time.

The graphical comparison between the raw OCT data and the corresponding longitudinal cross section of the welded seam is shown in Figure 3b. The black points show the raw OCT data. The actual weld depth, which was extracted from the longitudinal cross section, is marked by the blue line. Obviously, the measured points during the welding process were scattered over the measuring range and did not reveal the actual weld depth directly. Therefore, post-processing of the raw OCT data was required to extract the welding depth.

**Figure 4:**
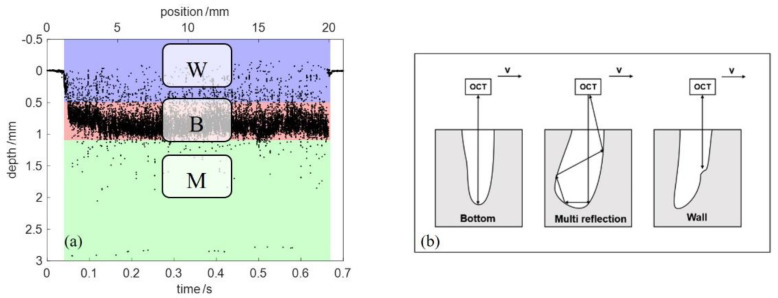
(**a**) Categorization of the raw OCT data into three regions: Wall, Bottom and Multi reflection. (**b**) Interaction of the OCT measuring beam with keyhole [27].

Since the fluctuation of the keyhole could be generated by process variation in the welding process, the OCT measuring beam through the keyhole was not necessarily reflected by the bottom of the keyhole. Change in the material thickness and material properties or welding of dissimilar materials will also affect the geometry of the keyhole. The measured points during the welding process can be categorized into three regions, as shown in Figure 4a. The dense measured points within the region B were the measurement of the keyhole bottom. The sparse measured points in the region M and W resulted from the multiple reflection and the keyhole wall. Figure 4b shows the interaction of the OCT measuring beam with the keyhole. The measured points in the region M and W can be viewed as outliers, which indicate the dynamic changes in the size and shape of the keyhole. These outliers will interfere with the subsequent extraction of the welding depth and need to be removed before applying any filtering methods. It should also be noted that both regions include very few noise points of the OCT imaging artifacts, which result from autocorrelations, speckle and white noise (such as detector noise and shot noise) [24,25,26].

### 2.4. Outlier Detection of the Raw OCT Data

**Figure 5:**
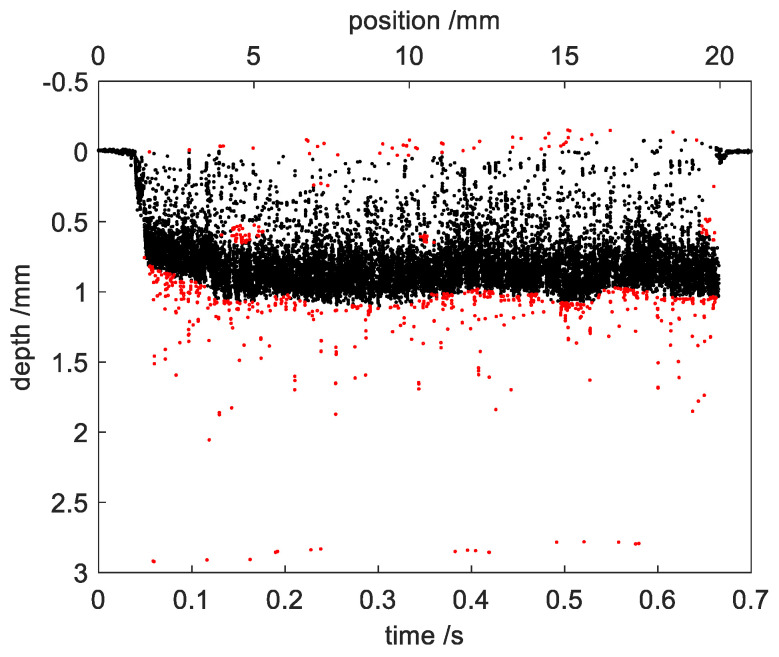
Outlier detection of the measured points during the welding process using LOF. The red points mark the detected outliers.

Outlier detection is usually used in the preprocessing of data analytic because the presence of outliers could affect the data analytic results. Outlier detection approaches can be classified into: statistical-based, distance-based, cluster-based and density-based [28]. As shown in Figure 4a, the density of the outliers within regions W and M was lower than that of the signals within region B. Based on the difference in density between signals and outliers, a density-based outlier detection method, LOF (local outlier factor) [29], was used to identify the outliers from the raw OCT data. The LOF computed the local density deviation of each data point with respect to its k nearest neighbors. A value of approximately 1 indicated the data point had similar density as its neighbors, while a value significantly larger than 1 meant that the data point had lower density than neighbors and can be considered as outliers. A threshold was set to identify the outliers. Figure 5 shows the outlier detection of the raw OCT data using LOF. Only the measured points during the welding process were included. A total of 100 nearest neighbors were considered and the threshold was set as 1.2. The red points mark the detected outliers. The outliers from the region M can be recognized completely, but in the region W, only part of the outliers were identified. The incomplete detection of outliers using LOF will affect the accuracy of the subsequent extraction of the welding depth.

To overcome the drawback of LOF, a cluster-based outlier detection method, DBSCAN [30], was applied to detect the outliers from the raw OCT data. DBSCAN can identify the noise in low density areas, which requires two user-defined parameters: radius of cluster (epsilon) and minimum of points (minPts). The input data point was labeled as:

1. Core point, if the number of points in an epsilon radius of it is not less than minPts;
2. Border point, if it is not a core point but within an epsilon radius of a core point;
3. Outlier, if neither a core point nor a border point.

**Figure 6:**
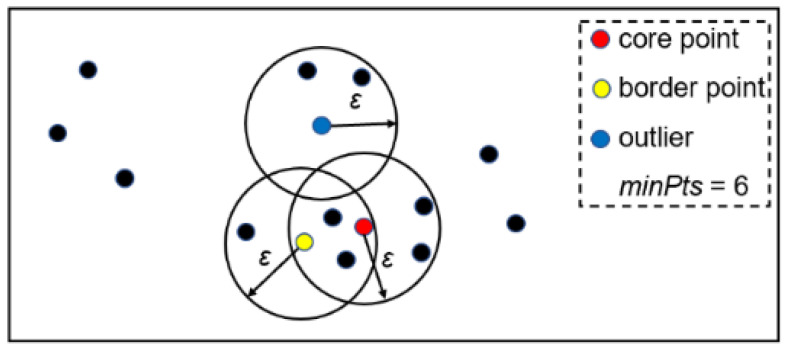
Schematic diagram of DBSCAN.

Figure 6 shows the schematic diagram of DBSCAN with minPts = 6. The red point was a core point because it contained six points in an epsilon radius. The yellow point was not a core point but within an epsilon radius of red point and, thus, belonged to a border point. The blue point was neither a core point nor a border point. The abstract DBSCAN algorithm is shown in Algorithm 1.
**Algorithm 1** Abstract DBSCAN Algorithm **Inputs**: dataset, epsilon, minPts  -Compute the points in the epsilon neighborhood of every point and identify core points  -Join neighboring core points into clusters  -**foreach** non-core point **do**  -   Add to a neighboring core point if possible  -   Otherwise, add to outlier **Output**: outliers


**Figure 7:**
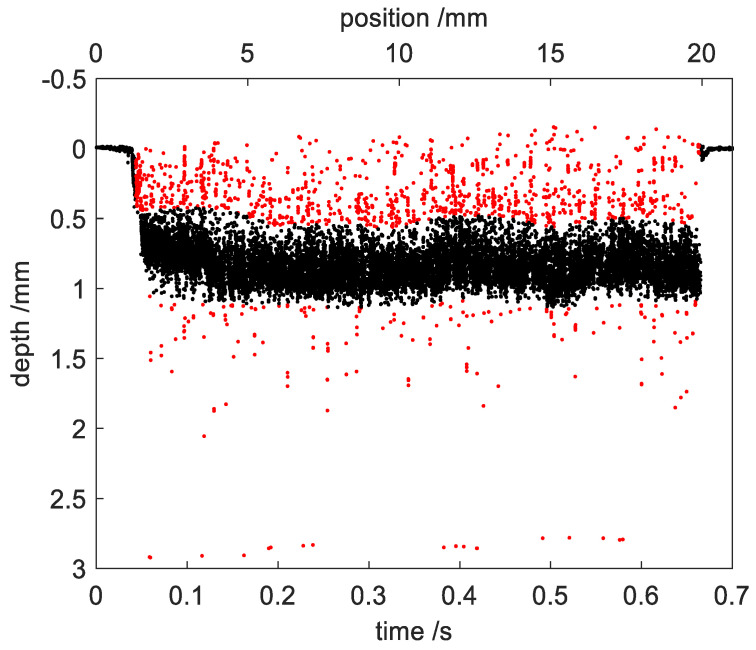
Outlier detection of the measured points during the welding process using DBSCAN. The red points mark the detected outliers.

Figure 7 shows the outlier detection of the measured points during the welding process using DBSCAN with epsilon = 0.05, minPts = 100. The black points show that the measured points within region B were clustered into one group. The red points mark the detected outliers. It can be seen that the outliers from both region M and W can be well separated from the measured points within region B.

### 2.5. Percentile Filter

To extract the welding depth, a filtering method needs to be applied to the OCT data. Compared with other filtering methods, percentile filter was confirmed to be viable to process the OCT data [20]. The percentile filter is a nonlinear statistical filtering technique [31], which requires two parameters: percentile *p* and window length *L*. The percentile filter works in a moving way with a centered window. The welding depth measurements within the window with a length of L are recorded at the times

(5) $$T_{i}=\left[t_{i-\frac{L}{2}+1},\cdots ,t_{i},\cdots ,t_{i+\frac{L}{2}}\right]$$

By applying the percentile filter with a percentile of *p*, the filtered depth can be expressed as

(6) $$D{\left(t_{i}\right)}_{p}=F_{p}\left[D\left(t\right),t\in T_{i}\right]$$

which means that *p*% of the depths are smaller than the filtered depth.

**Figure 8:**
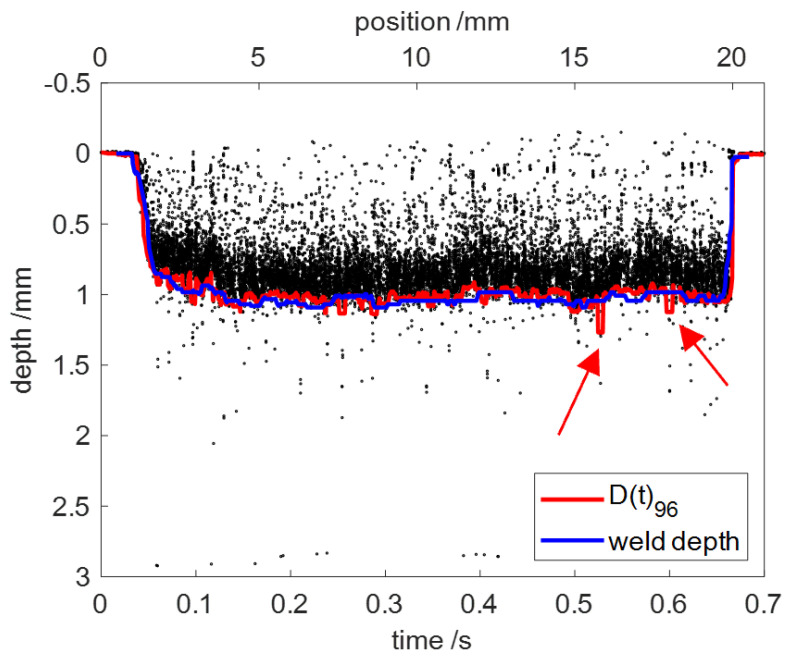
Welding depth extracted from the raw OCT-data using percentile filter (red) and the actual weld depth (blue). The red arrows mark the incorrect welding depth extraction.

Figure 8 shows the welding depth extracted from the raw OCT data using percentile filter with *p* = 96 and *L* = 200 in red. The actual weld depth was marked by blue as a reference. It can be seen that the accuracy of percentile filter for the extraction of the welding depth was easily affected by the outliers of the raw OCT data and it was, therefore, beneficial to remove the outliers before applying percentile filter.

To quantify the accuracy of the proposed method for the extraction of the welding depth, the difference Δd between the welding depth extracted from the OCT data and the actual weld depth extracted from the longitudinal cross section was considered. The average error was given by [21]

(7) $$E=\frac{\sum_{i=1}^{n}\left|\Delta d_{i}\right|}{n\cdot \bar{D}}$$

where n is the number of the considered measuring points, $\bar{D}$ is the average of the actual weld depth and only the measured points during the welding process are included.

## 3. Results and Discussion

### 3.1. Welding Depth Extracted from the Cleaned OCT Data

**Figure 9:**
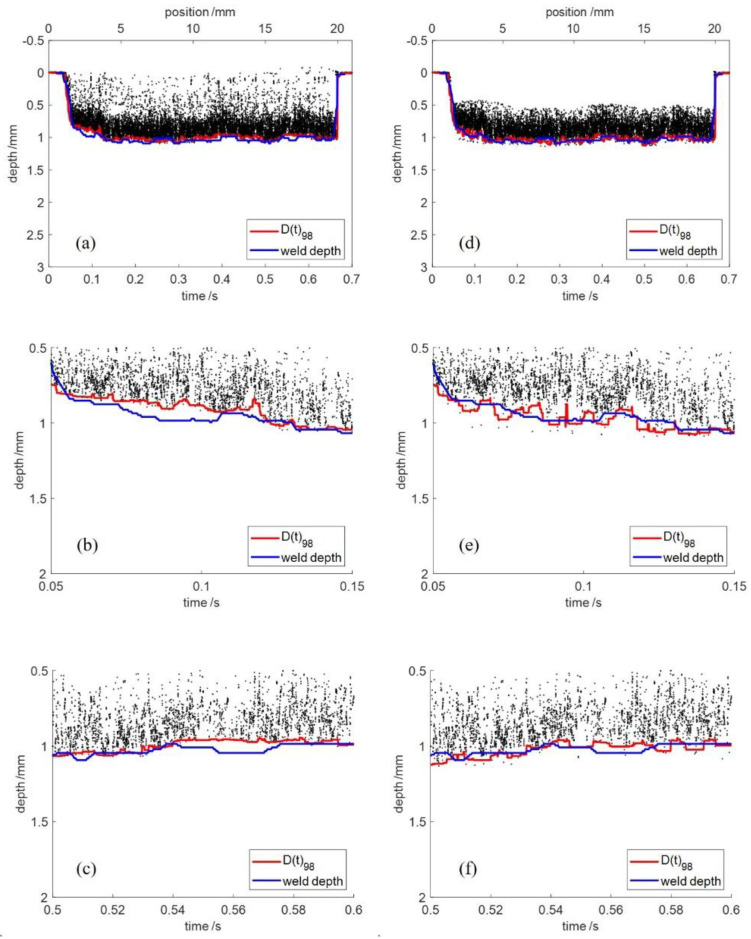
Welding depth extracted from the cleaned OCT data. (**a**–**c**) Outlier detection using LOF; (**d**–**f**) outlier detection using DBSCAN.

In this paper, the outliers of the raw OCT data were first detected using outlier detection methods. After eliminating the detected outliers, a percentile filter was applied to the cleaned OCT data to extract the welding depth. LOF and DBSCAN were applied to detect the outliers of the raw OCT data and same parameters of the percentile filter were used to extract the welding depth from the cleaned OCT data. The cleaned OCT data were obtained by LOF in Figure 9a and DBSCAN in Figure 9d. In both cases, the welding depth was extracted using percentile filter with *p* = 98 and *L* = 200. The welding depth extracted from the cleaned OCT data was marked in red and the actual weld depth extracted from the longitudinal cross section was marked in blue. Figure 9b,e shows the comparison of the time range of [0.05, 0.15] and Figure 9c,f shows the comparison of the time range of [0.5, 0.6]. It can be seen that the welding depth extracted by DBSCAN and percentile filter was more accurate than that extracted by the LOF and percentile filter.

The average error and processing time of the methods are shown in Table 2. In percentile filter, the average error was 4.4% and the processing time was 183 ms. In LOF and percentile filter, the average error was 3.9% and the processing time was 457 ms. In DBSCAN and percentile filter, the average error was 3.3% and the processing time was 581 ms. It can be seen that by applying outlier detection before percentile filter, the accuracy of welding depth extraction increased by up to 25%. In terms of the effectiveness of outlier detection, DBSCAN and percentile filter was better than LOF and percentile filter. The processing time of these two methods was longer than that of percentile filter.

### 3.2. Evaluation of the DBSCAN

**Figure 10:**
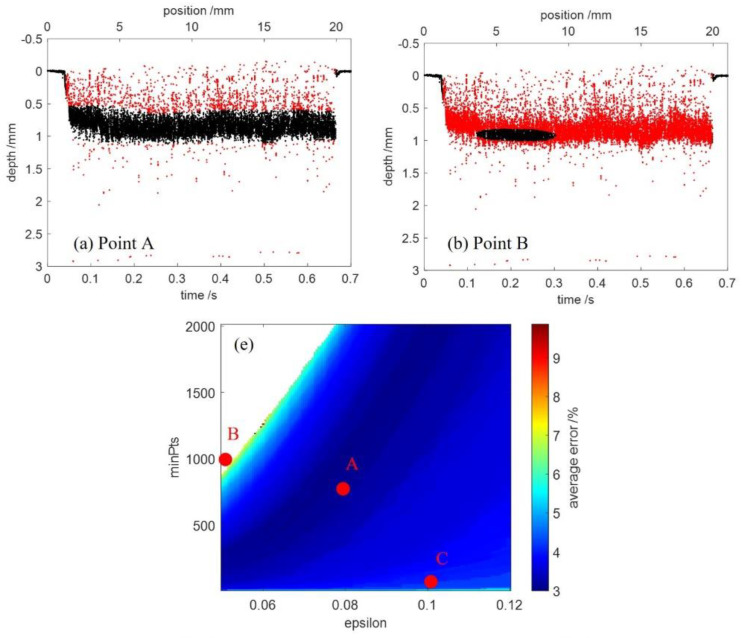

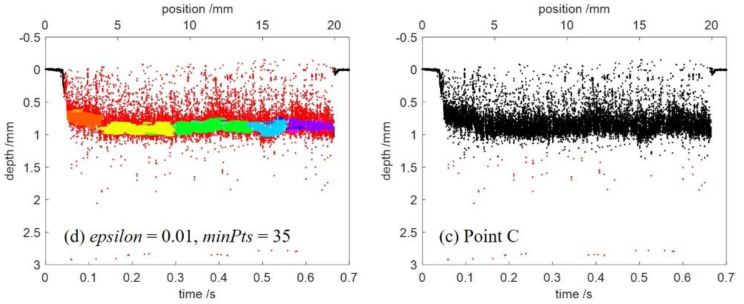
(**a**–**d**) Outlier detection of the measured points during the welding process using DBSCAN. The red points mark the detected outliers. The various colors in (**d**) show several groups of cluster. (**e**) Dependence of the average error in percent (color coded) on the epsilon and the minPts applied to DBSCAN. The parameters of DBSCAN are epsilon = 0.08, minPts = 750 for point A, epsilon = 0.05, minPts = 1000 for point B and epsilon = 0.1, minPts = 20 for point C.

In order to determine the best suited settings for DBSCAN, the average error (color coded) as a function of the epsilon and the minPts is shown in Figure 10e. The used percentile filter parameters were *p* = 98 and *L* = 200. The range of epsilon was [0.05, 0.12] at equal interval of 0.0005. The range of minPts was [10, 2010] at equal interval of 10. The average errors greater than 10% were eliminated, resulting in the blank space of the upper left corner.

The outlier detection using DBSCAN with epsilon = 0.08, minPts = 750 is shown in Figure 10a, where the signals were clustered into one group and the outliers of the raw OCT data can be detected completely. This case can be obtained over a broad range of the parameters and the average error was less than 5%. Figure 10b shows the outlier detection using DBSCAN with epsilon= 0.05, minPts = 1000. It can be seen that most of the signals were also detected as outliers when using parameters with low epsilon and high minPts. The outlier detection using DBSCAN with epsilon = 0.1, minPts = 20 is shown in Figure 10c, where outliers were detected incompletely. Figure 10d shows the outlier detection using DBSCAN with epsilon = 0.01, minPts = 35. In this case, the signals were clustered into several groups marked by various colors. It is advisable not to use parameters with epsilon as low as 0.01.

### 3.3. Evaluation of the Percentile Filter

**Figure 11:**
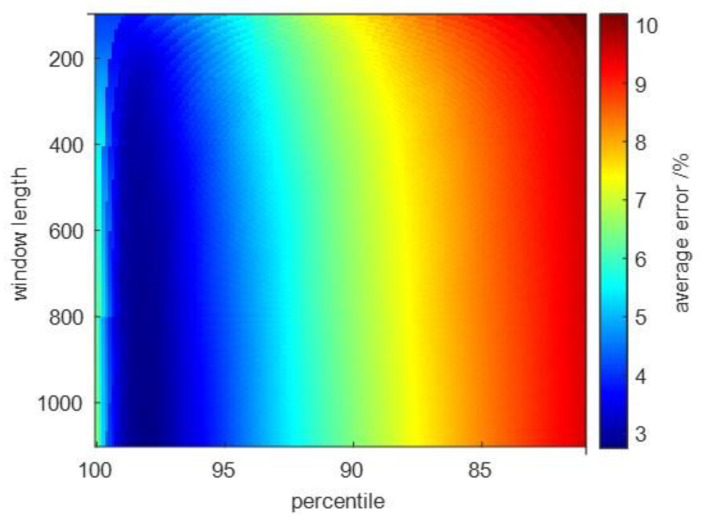
Average error E in percent (color coded) as a function of percentile *p* and window length *L* applied to percentile filter.

To determine the best parameters for the percentile filter, the dependence of the average error (color coded) on the percentile *p* and the window length *L* is shown in Figure 11. The used DBSCAN parameters were epsilon = 0.05, minPts = 100. It can be seen that a high percentile yielded smaller average error and the window length had minor influence on the average error. An average error of less than 5% can be achieved with a percentile between *p* = 96 and *p* = 99 and window length *L* ≥ 200.

### 3.4. Repeatability Validation

**Figure 12:**
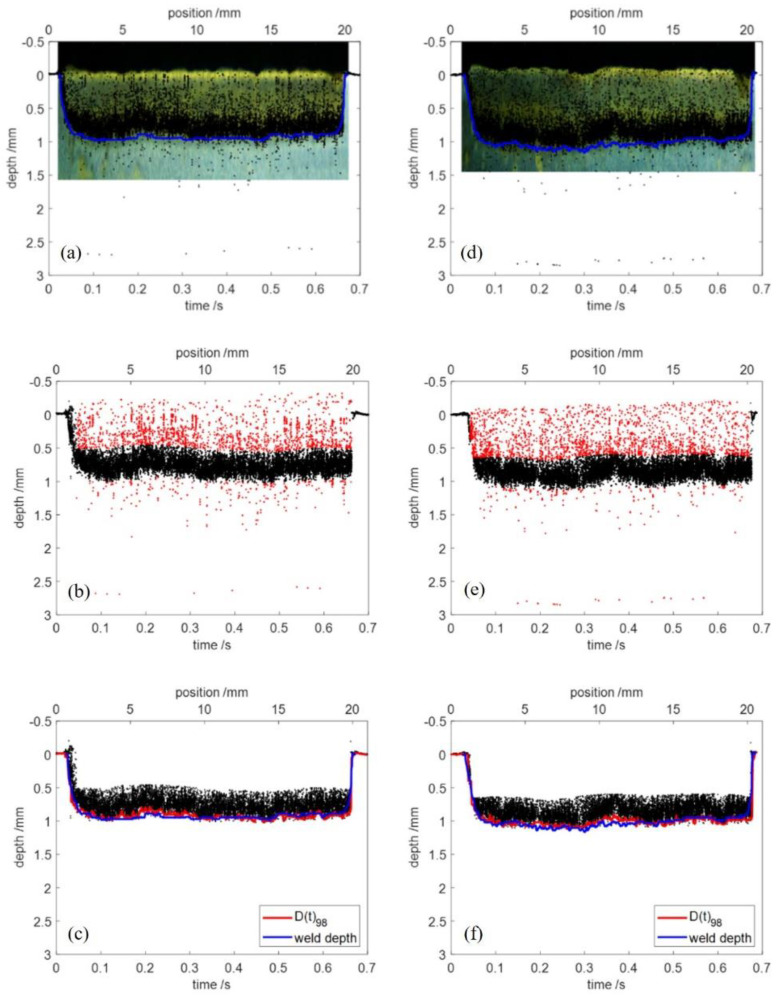
Two groups of experiments: (**a**–**c**) group 1; (**d**–**f**) group 2. The blue line in (**a**,**d**) marks the actual weld depth, which is extracted from the longitudinal cross section. The red points in (**b**,**e**) mark the detected outliers.

To further validate the repeatability of the proposed filter method, more than 10 groups of welds with the same process parameters were performed on mild steel with a thickness of 4 mm. The welding speed was set to 30 mm/s and the P/d to 2 kW/mm. Figure 12 shows two groups of these welds. Figure 12a,d shows the comparison of the raw OCT data and the actual weld depth extracted from the longitudinal cross section in blue. The outlier detection using DBSCAN are shown in Figure 12b with epsilon = 0.05, minPts = 200 and Figure 12e with epsilon = 0.1, minPts = 1000. Figure 12c,f show a comparison of welding depth extracted from the cleaned OCT data in red and the actual weld depth of longitudinal cross section in blue. In both cases, the used percentile filter parameters were *p* = 98 and *L* = 200. For all groups of experiments, the average error was less than 5%. The proposed method coupled DBSCAN and percentile filter removed the noise of the raw OCT data simply and extracted the welding depth from the raw OCT data precisely.

## 4. Conclusions

To eliminate the noise and further improve the extraction accuracy, this paper proposed an efficient method coupled DBSCAN and percentile filter for laser welding depth determination using optical coherence tomography. It was found that the noise of OCT data can be viewed as outliers and there was a significant difference in density between signals and noise. Density-based outlier detection approach DBSCAN was applied to the OCT data and the noise can be easily filtered out over a broad range of parameters. The percentile filter was then used to extract the welding depth from the cleaned OCT data. Using a percentile of 96–99 and a window length greater than 200, the extracted welding depth agreed well with the actual weld depth. The method efficiently extracted the laser welding depth with high-precision and will make the OCT technique more applicable for quality assurance in manufacturing production.

## Figures and Tables

**Table 1:** Chemical composition of Q235 mild steel.

Element	C	Si	Mn	P	S	Cr	Ni	Cu	Fe
**Mass fraction /%**	0.18	0.25	0.5	0.016	0.018	0.01	0.01	0.01	Others

**Table 2:** Average error and processing time of the methods.

	Percentile Filter	LOF + Percentile Filter	DBSCAN + Percentile Filter
Average error (%)	4.4	3.9	3.3
Processing time (ms)	183	457	581

## Data Availability

Not applicable.
